# Supplementary material for: Temporal mapping of the anti-tumor effects of nanobody-based MSLN.CAR-T cell therapy in metastatic solid tumors
Source: bioRxiv. 2025 Mar 2:2025.02.26.640438. Preprint. [Version 1] doi: 10.1101/2025.02.26.640438 (PMC12190766; doi:10.1101/2025.02.26.640438)
Supplement: Supplement 1 [file media-1.pdf]

Supp fig 1.

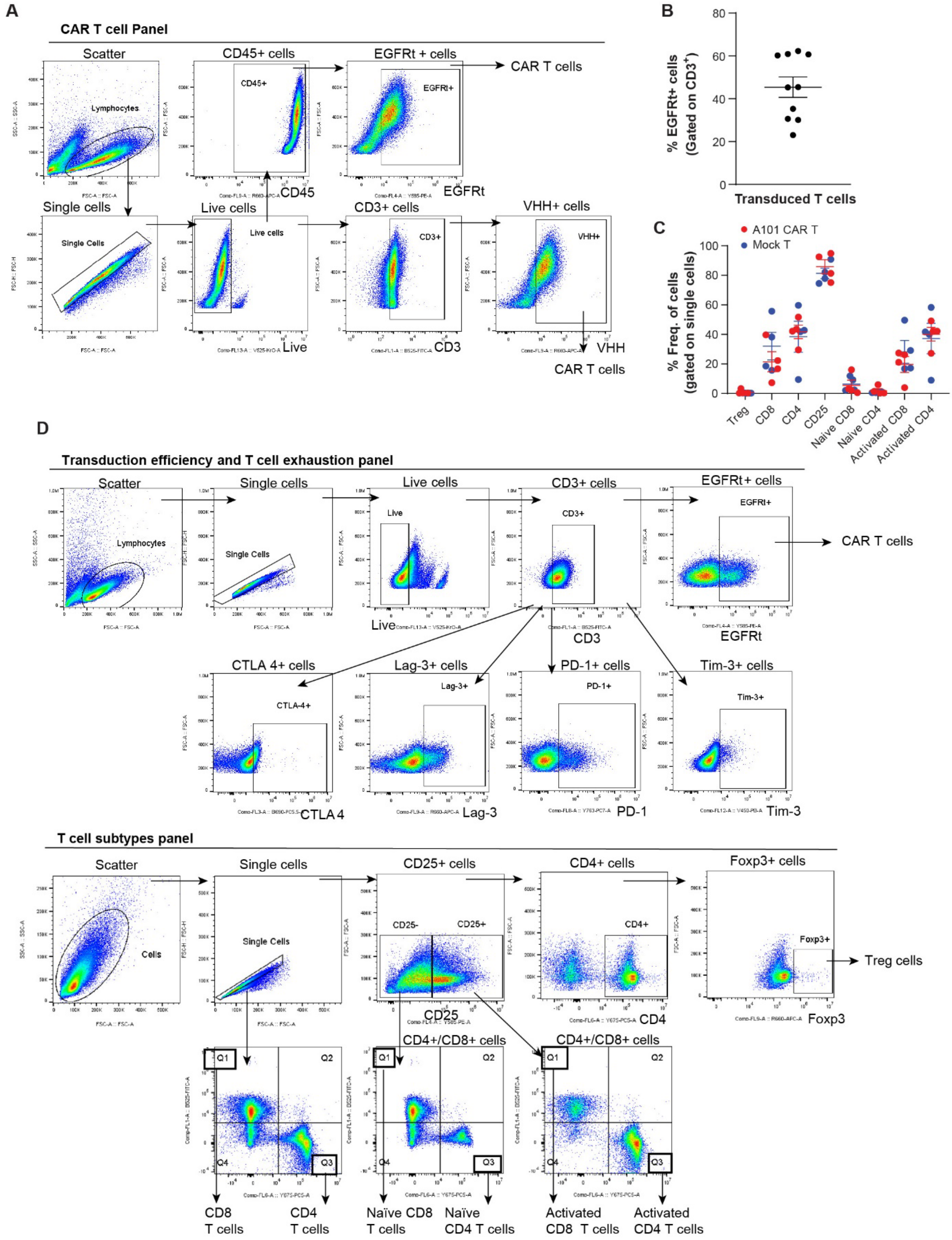
A
CAR T cell Panel
Scatter
Lymphocytes
CD45+ cells
CD45+
CD45
EGFRt + cells
EGFRt+
EGFRt
CAR T cells
Single cells
Single Cells
Live cells
Live
CD3+ cells
CD3+
CD3
VHH+ cells
VHH+
VHH
CAR T cells
B
% EGFRt+ cells (Gated on CD3+)
Transduced T cells
C
A101 CAR T
Mock T
% Freq. of cells (gated on single cells)
Treg
CD8
CD4
CD25
Naive CD8
Naive CD4
Activated CD8
Activated CD4
D
Transduction efficiency and T cell exhaustion panel
Scatter
Lymphocytes
Single cells
Single Cells
Live cells
Live
CD3+ cells
CD3+
CD3
EGFRt+ cells
EGFRt+
EGFRt
CAR T cells
CTLA 4+ cells
CTLA-4+
CTLA 4
Lag-3+ cells
Lag-3+
Lag-3
PD-1+ cells
PD-1+
PD-1
Tim-3+ cells
Tim-3+
Tim-3
T cell subtypes panel
Scatter
Cells
Single cells
Single Cells
CD25+ cells
CD25-
CD25+
CD25
CD4+ cells
CD4+
CD4
Foxp3+ cells
Foxp3+
Foxp3
Treg cells
CD4+/CD8+ cells
CD4+/CD8+ cells
Q1
Q2
Q3
Q4
CD8 T cells
CD4 T cells
Naïve CD8 T cells
Naïve CD4 T cells
Activated CD8 T cells
Activated CD4 T cells

Supplementary Fig. 1. A101 CAR-T cell product characterization *in vitro*. a Gating strategy for determining transduction efficiency. **b** Transduction efficiency at MOI 1.2 (n = 10 independent samples). Data points are mean ± SE. Gating strategy is shown in **d**. **c** The percentages of indicated immune cell subpopulations in total single cells quantified based on multi-parametric flow cytometry analysis on day 6 post-transduction. Data points are mean ± SE (n = 4 per group) and groups were compared using two-way ANOVA with Sidak's multiple comparisons tests (from left to right; F=0.09500, 1.286, 0.4011, 0.5651, 0.1145, 0.1430, 1.039, 0.3552, df=48 for all). No significant differences were found ($p \geq 0.05$). Gating strategy is shown in **d**.

Supp fig 2.

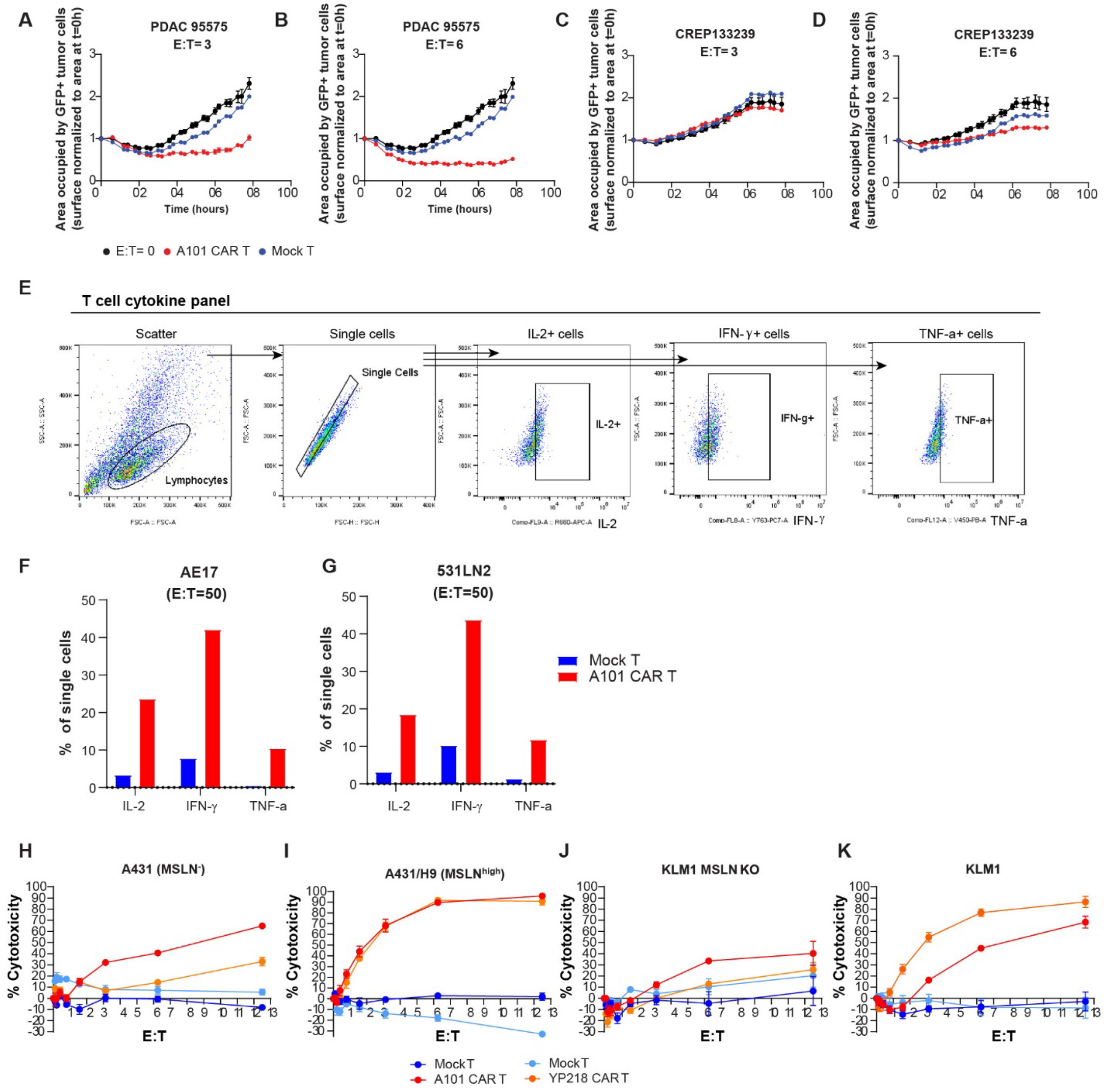

Supplementary Fig. 2. Cytotoxic activity of A101 CAR-T cells *in vitro*. a-d Cytotoxic activity of T cells during 78hrs of co-culture with GFP+ Msln+ murine tumor cell lines PDAC 95575 and CREP133239 at E:T ratio of 3 and 6 (n=3). Mean ± SE is shown. Groups were analyzed using two-way ANOVA with Tukey's multiple comparisons test (for PDAC 95575: F=464.7, df=6, for CREP133239: F=119.9, df=6). ****p<0.0001 **e** Gating strategy for detecting effector

cytokine expression. **f-g** Flow cytometric quantification of A101 CAR-T and mock-T cell expression of IFN-γ, IL-2 and TNF-α at E:T=50, 24hrs after coculture. n = 1. Gating strategy is shown in **e**. **h-k** Cytotoxic activity of human T cells when co-cultured with human tumor cells. The cell lines tested were A431 (**h**), A431/H9 (**I**), KLM1 MSLN KO (**j**) and KLM1 tumor cells (**k**). Data points are mean ± SE. Statistical analysis was performed using two-way ANOVA with Tukey's multiple comparisons test. For A431; n=2 independent experiments, A101 CAR-T vs mock-T; ****p<0.0001 (F=26.96, df=3). For A431/H9; n=4 (A101) and n=2 (YP218) independent experiments. A101 CAR-T vs mock-T; ****p<0.0001, YP218 CAR-T vs mock-T; ****p<0.0001 (F=83.24, df=3). For KLM1 MSLN KO; n=4 independent experiments, A101 CAR-T vs mock-T; ***p=0.0002, YP218 CAR-T vs mock-T; *p=0.0186 (F=8.851, df=3). For KLM1; n=6 independent experiments, A101 CAR-T vs mock-T; ****p<0.0001, YP218 CAR-T vs mock-T; ****p<0.0001 (F=54.99, df=3).

Supp fig 3.

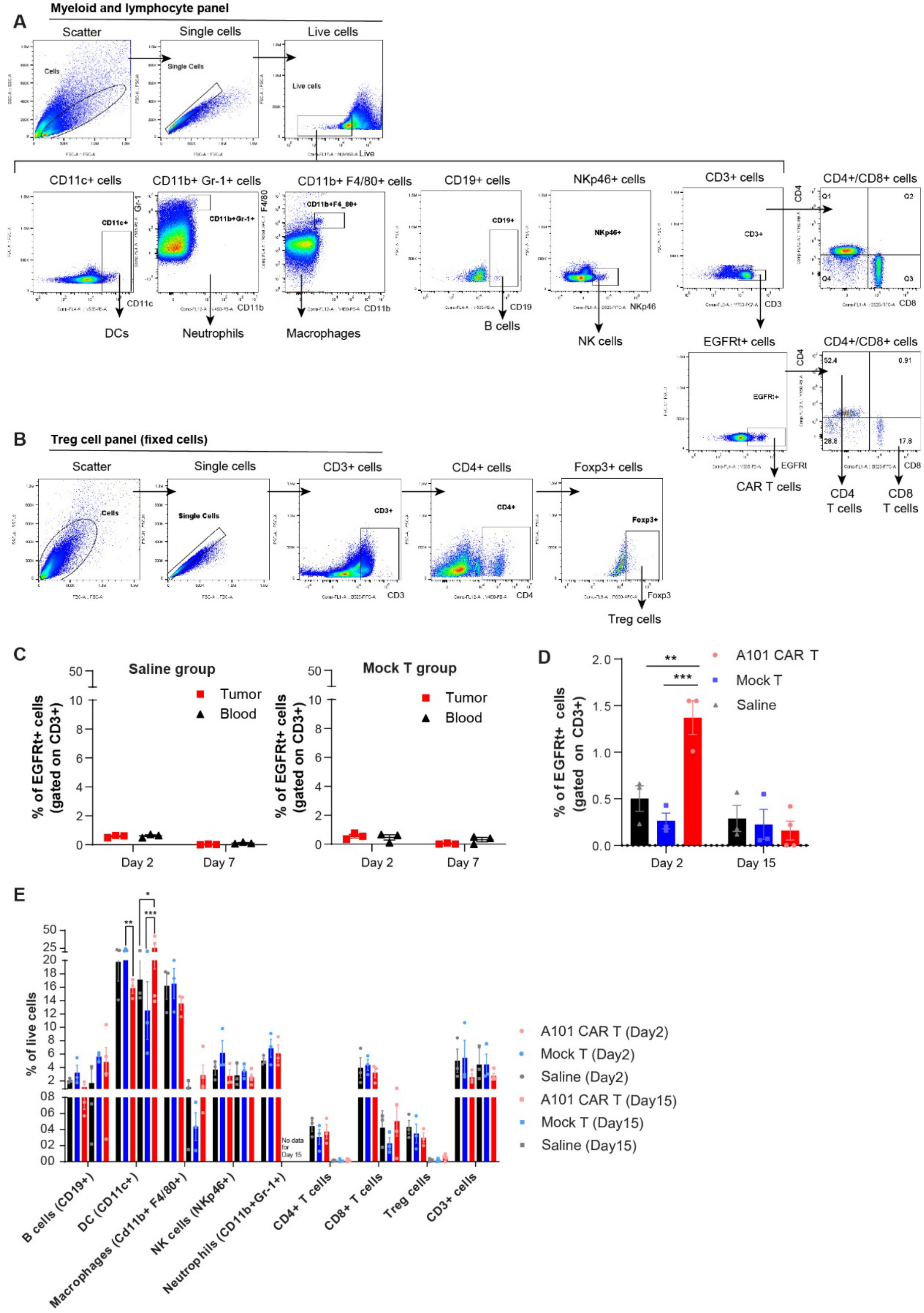

A
Myeloid and lymphocyte panel
Scatter
Single cells
Live cells
CD11c+ cells
CD11b+ Gr-1+ cells
CD11b+ F4/80+ cells
CD19+ cells
NKp46+ cells
CD3+ cells
CD4+/CD8+ cells
DCs
Neutrophils
Macrophages
B cells
NK cells
EGFRt+ cells
CAR T cells
CD4 T cells
CD8 T cells
B
Treg cell panel (fixed cells)
Scatter
Single cells
CD3+ cells
CD4+ cells
Foxp3+ cells
Treg cells
C
Saline group
Mock T group
Tumor
Blood
% of EGFRt+ cells (gated on CD3+)
Day 2
Day 7
D
A101 CAR T
Mock T
Saline
% of EGFRt+ cells (gated on CD3+)
Day 2
Day 15
E
% of live cells
A101 CAR T (Day2)
Mock T (Day2)
Saline (Day2)
A101 CAR T (Day15)
Mock T (Day15)
Saline (Day15)
No data for Day 15
B cells (CD19+)
DC (CD11c+)
Macrophages (Cd11b+ F4/80+)
NK cells (NKp46+)
Neutrophils (CD11b+Gr-1+)
CD4+ T cells
CD8+ T cells
Treg cells
CD3+ cells

Supplementary Fig. 3. Characterization of TME changes in response to A101 CAR-T cell treatment. a-b Gating strategy cell population analysis. **c** Percentage of $EGFRt^+$ cells among $CD3^+$ cells in tumors and blood of the control groups. **d-e** Treatment of PDAC 95575 syngeneic tumor bearing C57BL/6NCrL mice with 7 x 10^6 A101 CAR-T cells, mock-T cells or saline. Tumors were harvested on day 2 and 15 post treatment and characterized by flow cytometry. Live cells from tumor tissue were column sorted prior to flow cytometry. **d** The percentage of $EGFRt^+$ CAR-T cells among $CD3^+$ cells. Data points are mean ± SE (for day 2: n = 3, for day 15: n = 4 for CAR-T, n = 3 for mock-T and saline) and groups were compared using two-way ANOVA analysis with Tukey's multiple comparison tests (F=8.076, df=2). ***p=0.0002, **p=0.0019. **e** Cell population analysis of PDAC 95575 tumors. Data points are mean ± SE (for day 2: n = 3, for day 15: n = 4 for CAR-T, n = 3 for mock-T and saline) and groups were compared using two-way ANOVA analysis with Tukey's multiple comparison tests (day 2: F=7.059, df=2, day 15: F=1.424, df=2). **p=0.0011, ***p=0.0001, *p=0.0171. Gating strategy for (**d**) and (**e**) is shown in **a** and **b** respectively.

Supp fig 4.

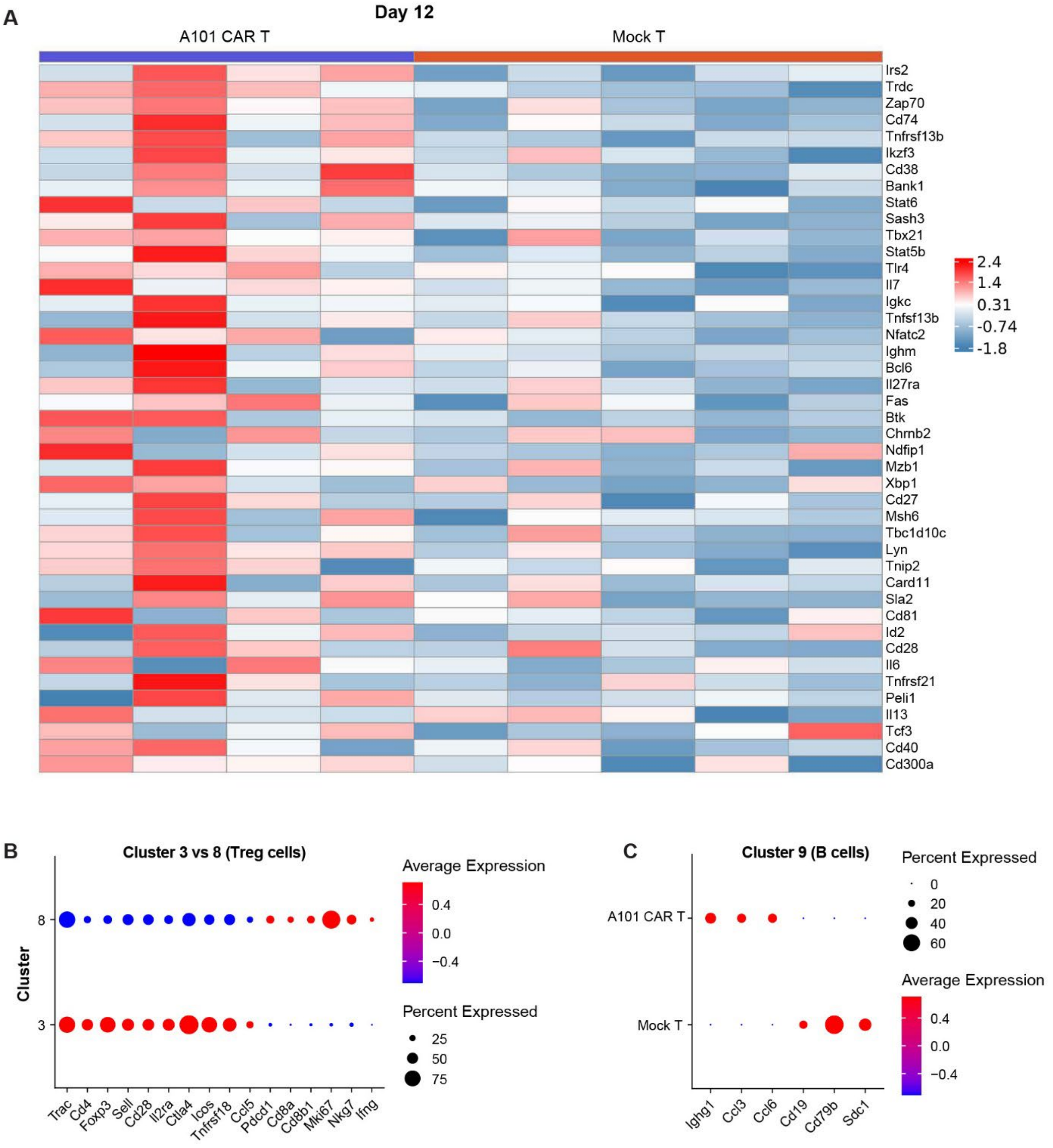

Supplementary Fig. 4. Total and scRNA-seq data analysis of 344SQ tumors. a Total RNA-seq data shows B cell activation on day 12 post treatment in A101 CAR-T-treated 344SQ tumors. Heat map of GSEA leading edge genes associated with the BP:GO_regulation_of_B_cell_activation pathway in A101 CAR-T- or mock-T-treated tumors on

day 12 post treatment (ES=0.44, NES=1.89, pval=0.00036, padj=0.014). **b-c** Dot plots visualize gene expression in indicated clusters. Red indicates a higher average expression of the gene, and a larger dot size indicates that the gene is present in a larger percentage of cells within that cluster. **b** scRNA FLEX-seq identifies two Treg cell subpopulations in 344SQ tumors. Scaled expression of significant, differentially expressed genes in the indicated clusters ($p<0.05$). **c** Comparison of Cluster 9 cells (B cells) between CAR-T and mock-T-treated 344SQ tumors. Scaled expression of significant, differentially expressed genes ($p<0.05$).

Supplementary Table 1. List of reagents and equipment.

Reagent or Resource	Source	Identifier
**Antibodies**		
Anti-mouse CD16/32 (Clone 93)	BioLegend	Cat#: 101302; RRID: AB_312801
A101 hFc	Dr. Mitchell Ho, NIH	N/A
PE goat anti-human IgG (polyclonal)	Jackson Research	Cat#: 109-116-170; RRID: AB_2337681
FITC anti-mouse CD3 (Clone 145-2C11)	Invitrogen	Cat#: MA5-17658; RRID: AB_2539048
PE anti-human EGFR (Clone Hu1)	R&D Systems	Cat#: FAB9577P; RRID: AB_2942015
APC anti-mouse CD45 (Clone I3/2.3)	BioLegend	Cat#: 147708; RRID: AB_2563539
iFluor 647 anti-camelid V_HH (Clone 96A3F5)	GenScript	Cat#: A01994; RRID: none
PE human IgG1 Isotype (Clone QA16A12)	BioLegend	Cat#: 403503, RRID: AB_3097044
eFluor 450 anti-mouse CD4 (Clone RM4-5)	eBioscience	Cat#: 48-0042-82, RRID: AB_1272194
PE/Cyanine5 anti-mouse CD4 (Clone GK1.5)	BioLegend	Cat#: 100409; RRID: AB_312694
FITC anti-mouse CD8 (Clone 53-6.7)	BioLegend	Cat#: 100706; RRID: AB_312745
PE anti-mouse CD25 (Clone PC61)	BioLegend	Cat#: 102007; RRID: AB_312856
APC anti-mouse Foxp3 (Clone FJK-16s)	BioLegend	Cat#: 17577382; RRID: AB_469457
PE/eFluor™ 610 anti-mouse CTLA-4 (Clone UC10-4B9)	eBioscience	Cat#: 61-1522-82; RRID: AB_2574581
APC anti-mouse Lag-3 (Clone eBioc9B7W)	eBioscience	Cat#: 17-2231-80; RRID: AB_2573183
PE-Cyanine7 anti-mouse PD-1 (Clone J43)	eBioscience	Cat#: 25-9985-80; RRID: AB_10853672
eFluor 450 anti-mouse Tim-3 (Clone 8B.2C12)	eBioscience	Cat#: 48-5871-80; RRID: AB_2574080

Supplementary Table 1. List of reagents and equipment.

APC anti-mouse IL-2 (Clone JES6-5H4)	Invitrogen	Cat#: 17-7021-81; RRID: AB_469489
PE-Cyanine7 anti-mouse IFN-γ (Clone XMG1.2)	Invitrogen	Cat#: 25-7311-82; RRID: AB_469680
eFluor 450 anti-mouse TNF-α (Clone MP6-XT22)	Invitrogen	Cat#: 48732180; RRID: AB_1548828
Anti-mouse CD3 (Clone CD3-12)	Bio-Rad Laboratories	Cat#: MCA1477; RRID: AB_321245
PE-Cyanine7 anti-mouse CD3 (Clone 17A2)	BioLegend	Cat#: 100219; RRID: AB_1732068
VioBlue anti-mouse CD11b (Clone M1/70.15.11.5)	Miltenyi	Cat#: 130-113-238; RRID: AB_2726047
PE anti-mouse CD11c (Clone N418)	Miltenyi	Cat#: 130-122-952; RRID: AB_2801981
PE anti-mouse CD19 (Clone 6D5)	Miltenyi	Cat#: 130–102-598; RRID: AB_2661112
APC anti-mouse F4/80 (REA126)	Miltenyi	Cat#: 130-116-525; RRID: AB_2733417
FITC anti-mouse NKp46 (Clone 29A1.4.9)	Miltenyi	Cat#: 130–102-300; RRID: AB_2661345
APCeF780 anti-mouse Gr-1 (Clone RB6–8C5)	Miltenyi	Cat#: 47–5931-80; RRID: AB_1518805
**Chemicals, peptides, recombinant proteins and assays**		
Dynabeads Mouse T-Activator CD3/CD28 for T-Cell Expansion and Activation	Gibco	Cat#: 11453D
Cell lysis buffer	Promega	Cat#: E1941
LIVE/DEAD™ Fixable Aqua Dead Cell Stain Kit, for 405 nm excitation	Invitrogen	Cat#: L34957
Foxp3/Transcription Factor Staining Buffer Set	eBioscience	Cat#: 00-5523-00
2β-mercaptoethanol	Sigma-Aldrich	Cat#: M6250
DNase I recombinant, RNase-free	Roche	Cat#: 10104159001
Fetal Bovine Serum	GemCell	Cat#: 100-500
Penicillin-Streptomycin	Gibco	Cat#: 15140-122
L-Glutamine	Gibco	Cat#: A2916801

Supplementary Table 1. List of reagents and equipment.

Sodium Pyruvate	Sigma-Aldrich	Cat#: S8636
DMEM, high glucose, GlutaMAX™ Supplement	Gibco	Cat#: 10566016
BD Cytofix/Cytoperm™ Fixation/Permeabilization Kit	BD Pharmingen	Cat#: 554714
Human IL-2	Prometheus Therapeutics & Diagnostics	Cat#: NDC 65483-116-07
Mouse IFN-gamma Quantikine ELISA Kit	R&D Systems	Cat#: MIF00
RNaseZap™ RNase Decontamination Solution	Invitrogen	Cat#: AM9782
RNAlater™ Stabilization Solution	Invitrogen	Cat#: AM7021
RBC lysis buffer	BioLegend	Cat#: 420301
Non-essential Amino Acid Solution	Sigma-Aldrich	Cat#: M7145
EDTA	Sigma-Aldrich	Cat#: E7889
BSA	Cell Signaling	Cat#: 9998s
Liberase	Sigma-Aldrich	Cat#: 5401020001
Lentiblast	OzBiosciences	Cat#: LBPX1500
MycoAlert® Mycoplasma Detection Kit	Lonza	Cat#: LT07-318
Luciferase Assay System	Promega	Cat#: E1501
Pan T cell isolation kit II, mouse	Miltenyi	Cat#: 130-095-130
Leica Bond Polymer Refine Kit	Leica Biosystems	Cat#: DS9800
OneComp eBeads™ Compensation Beads	ThermoFisher Scientific	Cat#: 01-1111-42
CD45 (TIL) MicroBeads, mouse	Miltenyi	Cat#: 130-110-618
Dead cell removal kit	Miltenyi	Cat#: 130-090-101
Chromium Next GEM Single Cell Fixed RNA sample preparation kit	10x Genomics	Cat#: 1000414
RNeasy Mini Kit	Qiagen	Cat#: 74104
ArC™ Amine Reactive Compensation Bead Kit	Invitrogen	Cat#: A10346
DAPI	Invitrogen	Cat#: D1306
Zombie UV™ Fixable Viability Kit	BioLegend	Cat#: 423107
LS columns	Miltenyi	Cat#: 130-042-401
MS columns	Miltenyi	Cat#: 130-042-201
**Cell lines & mouse strains**		
Mouse: PDAC 95575	Dr. Serguei Kozlov, CAPR, NIH	N/A
Mouse: PDAC 95575 GFP+/Luc+	Dr. Mitchell Ho, NIH	N/A
Mouse: 344SQ	Jonathan M. Kurie, MD Anderson Cancer Center	N/A

Supplementary Table 1. List of reagents and equipment.

Mouse: AB12	Sigma-Aldrich	Cat#: 10092306-1VL
Mouse: AE17	Sigma-Aldrich	Cat#:10092310-1VL
Mouse: Panc02	Cytion	Cat#: 300501
Mouse: 531LN2	Jonathan M. Kurie, MD Anderson Cancer Center	N/A
Mouse: TC-1	Prof. TC.Wu, Department of Pathology, SOM, JHU (NCI MTA. #49891-22)	N/A
Mouse: CREP133239 GFP+/Luc+	Dr. Mitchell Ho, NIH	N/A
Mouse: LLC (LL/2 (LLC1))	ATCC	Cat#: CRL-1642
Human A431	Ira Pastan, NCI	N/A
Human: A431/H9	Ira Pastan, NCI	N/A
Human: KLM1	Christine Alewine, NCI	N/A
Human: KLM1 MSLN KO	Christine Alewine, NCI	N/A
Mouse: 129S2/SvPasCrl	Charles River	Strain# 476
Mouse: C57BL/6NCrL	Charles River	Strain# 027
Mouse: B6.SJL-PtprcaPepcb/BoyCrCrl	Charles River	Strain# 564
**Recombinant DNA**		
GFP/Luciferase transduction: R980-M03-663 mPol2 ffLuc2-eGFP (pFUGW)	NCI, Frederick, NIH	N/A
Human A101 CAR plasmid	Dr. Mitchell Ho, NIH	N/A
Mouse A101 CAR plasmid	Dr. Chaido Stathopoulou, NIH	N/A
**Software and algorithms**		
GraphPad PRISM 9	GraphPad	RRID:SCR_002798
FlowJo (version 10.7.2)	FlowJo LLC.	RRID:SCR_008520
CytoFLEX flow cytometer	Beckman Coulter, USA	RRID:SCR_025068
BioRender	BioRender	RRID:SCR_018361
Halo	Indica Labs	RRID:SCR_018350
Aperio ImageScope	Leica Biosystems	RRID:SCR_020993
Seurat (version 4.4.0)	Seurat	RRID:SCR_007322
R	R	RRID:SCR_001905
STAR	STAR	RRID:SCR_004463
RSEM	RSEM	RRID:SCR_000262
LIMMA	LIMMA	RRID:SCR_010943

Supplementary Table 1. List of reagents and equipment.

CIBERSORT	Stanford University; Stanford; California	RRID:SCR_016955
